# Impact of physician−pharmacist collaborative protocol-based pharmacotherapy management for HIV outpatients: a retrospective cohort study

**DOI:** 10.1186/s40780-020-00165-9

**Published:** 2020-05-01

**Authors:** Kimihiko Urano, Miki Ishibashi, Takeshi Matsumoto, Kohshi Ohishi, Yuichi Muraki, Takuya Iwamoto, Junichi Kunimasa, Masahiro Okuda

**Affiliations:** 1 Department of Pharmacy, Mie University Hospital, 2-174, Edobashi, Tsu, Mie 514-8507 Japan; 2 Department of Clinical Pharmacokinetics, School of Pharmacy, Aichi Gakuin University, 1-100, Kusumoto-cho, Chikusa-ku, Nagoya, Aichi 464-8650 Japan; 3 Transfusion Medicine and Cell Therapy, Mie University Graduate School of Medicine, 2-174, Edobashi, Tsu, Mie 514-8507 Japan; 4 Department of Clinical Pharmacoepidemiology, Kyoto Pharmaceutical University, 5, Misasagi-Nakauchi-cho, Yamashina-ku, Kyoto, 607-8414 Japan; 5 Education and Research Center for Clinical Pharmacy, Kobe Pharmaceutical University, 4-19-1, Motoyamakitamachi, Higashinada-ku, Kobe, Hyogo 658-8558 Japan; 6 Department of Pharmacy, Osaka University Hospital, 2-15 Yamadaoka, Suita, Osaka, 565-0871 Japan

**Keywords:** HIV, AIDS, PBPM, Pharmacist

## Abstract

**Background:**

Effective treatment for human immunodeficiency virus (HIV) infection requires close cooperation among healthcare professionals. This is because maintaining continuity with treatment regimens is important in anti-HIV therapy. In addition, explaining medication use is more important than that for other diseases. Since 2010, pharmacists at the Mie University Hospital have been interviewing patients, selecting drugs, and formulating medication plans for HIV-positive patients. In August 2011, we established the physician and pharmacist-led collaborative Protocol-based Pharmacotherapy Management (PBPM) to increase the efficacy and safety of treatment, while reducing the burden on physicians. In the present study, we evaluated the outcomes associated with PBPM for HIV pharmacotherapy.

**Methods:**

We prepared protocols for drug selection, timing of interventions, and methods of intervention according to various guidelines. This study included 40 HIV-positive patients receiving outpatient care between January 2009 and February 2017. Of these patients, 17 received treatment before implementing PBPM and 23 patients received treatment afterward. We compared the intervention parameters between before and after the implementation of PBPM.

**Results:**

The proportion of patients receiving prescription proposals from pharmacists was markedly higher after introducing PBPM (6 out of 17 patients vs. 23 out of 23 patients). All prescription proposals were accepted by physicians before and after PBPM. The number of interviews before antiretroviral therapy (ART) initiation (median [range]) decreased from 2 [1–5] to 1 [1–3] after PBPM introduction, suggesting the time to introduction of treatment has been shortened. Before the introduction of PBPM, nine patients required a change in their ART prescriptions and four patients were hospitalized (one patient was hospitalized due to an error in the self-administration of anti-HIV medicines, two patients were hospitalized due to interruptions in medication, and one patient was hospitalized for the treatment of other diseases). Only one patient was hospitalized after PBPM, and was unrelated to drug adherence. The proportion of patients with a reduced HIV-RNA load increased from 71 to 100%. Furthermore, the proportion of patients who maintained levels below the limit of quantitation increased from 59 to 91% after implementing PBPM.

**Conclusion:**

The implementation of PBPM for HIV outpatients improves the efficacy and safety of HIV pharmacotherapy.

## Background

Antiretroviral therapy (ART) has markedly improved the prognosis of human immunodeficiency virus (HIV) infection. Furthermore, understanding the importance of continuous medication adherence is essential for successful treatment over the patient’s lifespan. Adverse reactions due to mistakes in self-administration lead to crucial problems. Therefore, it is necessary to carefully monitor patient adherence, which requires not only consulting physicians but cooperating with professionals involved with HIV pharmacotherapy.

In the United States, pharmacists are legally permitted to order clinical tests, administer medications, and monitor patients according to the Collaborative Drug Therapy Management (CDTM) agreements with doctors [1]. CDTM collaborations have spread throughout the country with demonstrated clinical efficacy. Pharmacists in the United States and Australia also actively participate in treating HIV patients, thus improving treatment outcomes and reducing medical expenses [2, 3]. However, Japan is legally prohibited from operating this type of system. Therefore, the PBPM was established as an alternative system where pharmacists participate in treatments based on protocols that are suggested by physicians.

Actively involving pharmacists in HIV treatment has been shown to improve patient counts of CD4-positive lymphocytes, which is a key indicator of outpatient drug-related therapeutic efficacy [4]. It has also been reported that ART-related medication errors are reduced by involving pharmacists, even if these errors occur with hospitalized patients [5]. Currently, a Protocol-based Pharmacotherapy Management (PBPM) plan based on CDTM, in which pharmacists conduct interventions based on a doctor-approved protocol, is being introduced in Japan. According to the PBPM rubric in Japan, pharmacists are now participating in anti-HIV therapy [6]. However, there have been no reports on whether the efficacy or outcomes of HIV are affected by this scheme.

At Mie University Hospital, we established a team-based system for outpatient anti-HIV therapy in 2010. Under this system, pharmacists participate in patient interviews and outpatient guidance to select the appropriate ART and formulate a treatment regimen for HIV treatment. However, hospitalization was still required in some cases due to acute renal failure caused by overdose or adverse reactions triggered by the patient discontinuing medications. These adverse outcomes were attributed to a lack of involvement by pharmacists. In August 2011, we established a doctor−pharmacist PBPM aimed at improving the efficacy and safety of anti-HIV treatment and reducing the burden on doctors. In this study, we evaluated the efficacy of this PBPM for anti-HIV drug therapy.

## Methods

### Clinical protocol

**Fig. 1:**
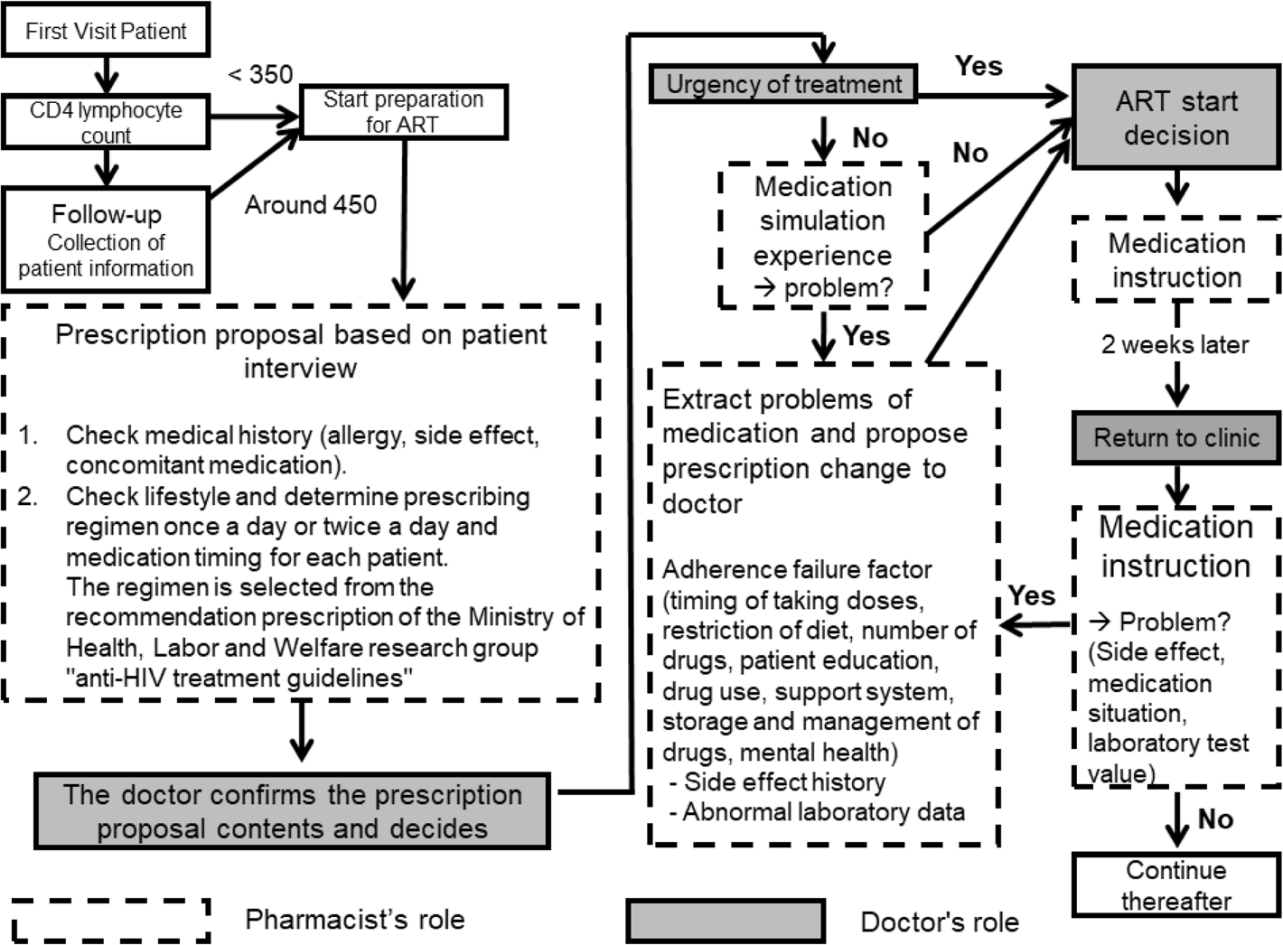
PBPM flowchart for anti-HIV therapy

The study subjects were HIV patients in the Department of Blood Medicine outpatient clinic at the Mie University Hospital in Tsu, Japan. The treatment protocol, which involved the selection of appropriate drugs, determining the timing of intervention, and the method of intervention, was based on HIV treatment guidelines and approved by medical specialists in blood medicine. All protocols were updated according to the revised guidelines (Fig. 1).

### Subjects

Forty patients treated at the HIV outpatient clinic between January 2009 and February 2017 were enrolled (17 patients were treated before the implementation of PBPM while 23 were treated after the introduction of PBPM; Table 1). This study was an observational study, and all patients who received medical treatment during the observation period were enrolled in this study. All subjects had either recently began ART or changed their ART medications during the study period. The following endpoints were evaluated: the number of prescription proposals written by pharmacists, the number of interviews required before beginning ART, the number of patients who changed prescriptions within 6 months (as well as their reasons for changing medication), the proportion of patients with reduced HIV-RNA, and the percentage of patients maintaining HIV-RNA levels below the limit of quantitation. A decrease in HIV-RNA level was defined as reaching the detection limit below 6 months after the start of ART administration. In addition, maintaining below the detection limit was defined as maintaining the detection limit for 1 year after the start of ART administration.

**Table 1:** Patient demographics and medical history

	Before PBPM		After PBPM		*p*-value
Number of patients	17		23		
Other infectious diseases	HBV co-infection	2	HBV co-infection	5	
	HCV co-infection	0	HCV co-infection	2	
	Syphilis infection	4	Syphilis infection	5	
Nationality (M/F)	Japanese (9/1) 58%		Japanese (18/0) 78%		
	Other (2/5) 41%		Other (4/1) 22%		
Age	43 [30–59]		38 [20–69]		0.237
ART regimen	LPV/r + TDF/FTC	6	DRV + RTV + TDF/FTC	7	
	RAL + TDF/FTC	4	EVG/cobi/TDF/FTC	5	
	EFV + TDF/FTC	3	DTG + TDF/FTC	5	
	DRV + RTV + TDF/FTC	2	RAL + TDF/FTC	3	
	other	2	other	3	
HIV-RNA level at the start of treatment (copy/mL)	27,400 [N.D.–349,000]		44,900 [N.D.–100,000,000]		0.330

Median [range]

*LPV/r* Lopinavir/ritonavir; *TDF* Tenofovir disoproxil fumarate; *FTC* Emtricitabine; *RAL* Raltegravir;

*EFV* Efavirenz; *DRV* Darunavir; *RTV* Ritonavir; *EVG* Elvitegravir; *cobi* Cobicistat; *DTG* Dolutegravir

These endpoints were compared between patients treated during the pre- and post-PBPM periods.

### Statistical analysis

Patient background variables were compared using the Chi-Square (χ^2^) test and survey answers were compared using Fisher’s exact test. The significance level was set at *p* < 0.05 (one-sided) for all tests.

## Results

Before implementation of PBPM, pharmacists performed interviews voluntarily at the request of physicians. After the introduction of PBPM, the pharmacist interviewed all patients who were starting ART therapy. The contents of interviews with pharmacists were standardized by unifying question items. In addition, cooperation based on the consensus of physicians and pharmacists was conducted in accordance with the protocol (Table 2).

**Table 2:** Comparison of pharmacist’s role between before and after PBPM implementation

	Before PBPM	After PBPM
Opportunities for interviews with pharmacists	Voluntary	Mandatory (before and after ART administration)
Pharmacist interview contents	Not standardized	Standardized (Lifestyle, medication timing, etc.)
Collaboration between physicians and pharmacists	Pharmacist suggestions are not based on physician-pharmacist consensus	Consensus-based cooperation

Of the 17 cases reviewed before the introduction of PBPM, six patients (35%) received a prescription proposal written by a pharmacist to physicians, whereas all 23 cases interviewed by pharmacists after the introduction of PBPM received such proposals. There was no significant difference in the acceptance rate before and after the implementation of HIV-PBPM. During both periods, all suggestions were accepted and implemented. The number of interviews with patients conducted before beginning ART (median [range]) decreased from two (1−5) before PBPM to one (1−3) after the introduction of PBPM (Table 3).

**Table 3:** Number of prescription proposals and interviews by pharmacists before and after PBPM implementation

	Before PBPM	After PBPM
Number of patients	17	23
Number of patients receiving pharmacist intervention	6	23
Prescriptions proposed (accepted)	10 (10)	26 (26)
Number of interviews required to introduce ART	14	32
Number of interviews per patient to introduce ART	2 [1–5]	1 [1–3]

Median [range]

**Fig. 2:**
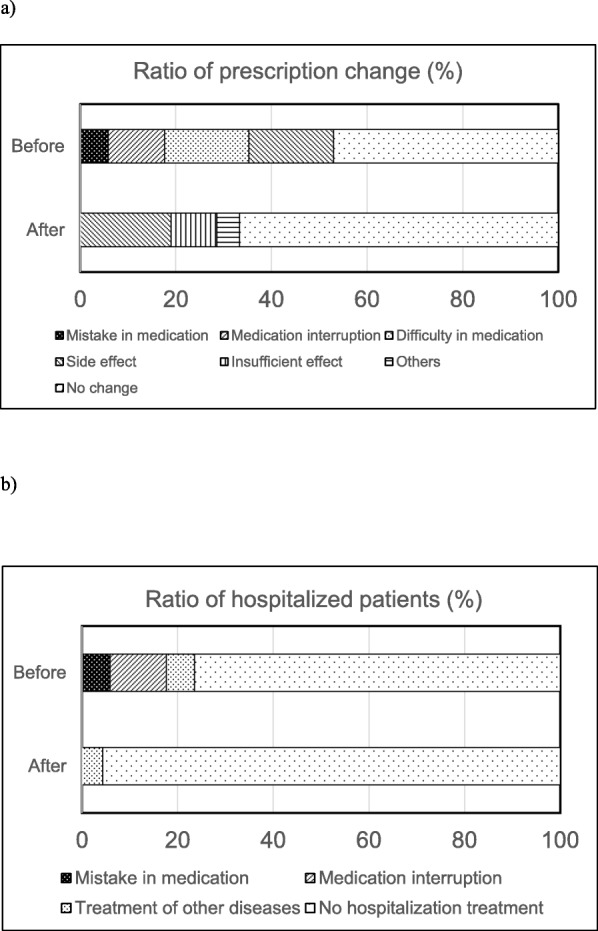
Comparison of ART treatment outcome before and after PBPM. a) Number of patients receiving ART drug change and the reasons. b) Number of patients requiring hospitalization and the reason

Before the implementation of PBPM, nine out of 17 patients received prescription changes, and four of these patients required hospitalization. One hospitalization was due to an error made during the self-administration of anti-HIV medicines, two patients were hospitalized for interruptions in medication use, and one patient was hospitalized for the treatment of other diseases. The most common reasons for hospitalization were kidney disorder followed by overdose or relapse with non-compliance. Pharmacists were not involved in any of these cases except during the time that they dispensed medications to patients. After the introduction of PBPM, only one patient required hospitalization for the treatment of other diseases, while no patients were admitted due to the inappropriate use of ART. In summary, there were fewer hospital admissions following the introduction of PBPM despite an increase in the number of prescription changes (Fig. 2). Moreover, the proportion of patients exhibiting reduced HIV-RNA levels increased from 71 to 100% after the introduction of PBPM. Also, the proportion of patients with HIV-RNA levels below the limit of quantitation also increased from 59 to 91% (Table 4).

**Table 4:** Comparison of therapeutic effects of ART before and after PBPM

	Before PBPM	After PBPM	*p*-value
Number of patients	17	23	
Number of patients with decreased HIV-RNA levels (percentage of total patients, %)	12 (71)	23 (100)	0.009
Number of patients with maintained HIV-RNA level below the detection limit (percentage of total patients, %)	10 (59)	21 (91)	0.023

## Discussion

The benefits of using PBPM have been well-reported. For instance, the frequency of severe cases of gastrointestinal bleeding was reduced by pharmacists during the administration of drugs for stress ulcer prophylaxis according to the protocol for preventing stress ulcers in the intensive care unit [7]. Similarly, in the current study, the frequency of adverse events associated with poor adherence to ART decreased after the implementation of PBPM (Fig. 2). Some of the adverse events occurring before the implementation of HIV-PBPM were caused by mistakes made by patients during the self-administration of anti-HIV medications. After the implementation of PBPM, no such cases occurred. These adverse events included renal dysfunction due to an overdose of anti-HIV medicines and relapse due to patient non-compliance.

The number of interviews conducted with patients after the implementation of PBPM decreased compared to the number of interviews before PBPM (Table 2). This result suggests that pharmacist’s intervention reduced the delay in therapeutic decisions. Therefore, intervention by pharmacists based on HIV-PBPM appears to improve the safety of medical care for outpatient HIV treatment. This finding is in agreement with a previous study showing that intervention by pharmacists in anti-HIV therapy reduces the frequency of adverse events in Brazilian hospital settings [8] .

The involvement of pharmacists appeared to not only decrease the frequency of adverse events but also improved treatment efficacy as evidenced by the reduction in the HIV-RNA levels. Similarly, a previous study [9] showed that pharmacist involvement improved therapeutic efficacy as evidenced by the lower number of CD4-positive lymphocytes and reduced rate of superinfection (co-infection) within 6 months. In this study, pharmacists were able to provide a variety of patient care services as part of a physician-pharmacist collaborative protocol. Specifically, pharmacists were able to order laboratory tests to monitor the safety and effectiveness of anti-HIV medication and treat adverse effects such as nausea and diarrhea.

Since this study is a retrospective comparison between two sequential time periods (before and after the implementation of PBPM), it is conceivable that other improvements in HIV treatment may account for these differences in safety and outcomes. For example, a preparation requiring only one tablet per day has been developed for better patient adherence. It has been reported that decreasing the number and frequency of medications improves adherence to treatment regimens [10]. HIV guidelines also suggest improving adherence using a single-agent regimen. It was reported that adherence and rates of viral suppression increase as the number of oral tablets decreases [11]. Although we did not examine patient adherence, it is generally believed that the reduction in levels of HIV-RNA reflects patient adherence and thus may be used as an alternative indicator. In the present study, the HIV-RNA load decreased, but no significant difference was observed between the number of doses per day used before and after the implementation of PBPM (1.8 ± 0.4 before implementation vs. 1.5 ± 0.5 after implementation; *p* = 0.936). However, there was a reduction in the number of agents used after the implementation of PBPM (3.9 ± 1.4 before implementation vs. 2.8 ± 1.5 after implementation; *p* = 0.027). The combination single-tablet has been commercially available since 2013. In addition, the average number of agents used for anti-HIV treatment prior to the sale of the combination tablets was 4.0 ± 0.9 agent (*n* = 11), and the number of agents did not change compared to the numbers before the introduction of PBPM. The reduction in the frequency of medication-related adverse events suggests that an appropriate regimen under PBPM could contribute to improved safety.

Currently, hospital pharmacists participate in the implementation of ART. However, not all outpatients receive services from pharmacists in outpatient clinics. On the other hand, community pharmacists who prescribe medications to HIV patients have the opportunity to monitor patient adherence and adverse events related to anti-HIV medicines. Improved cooperation between hospital pharmacists and community pharmacists may be insufficient to determine patient conditions. Therefore, we propose that it is still necessary to involve professionals working outside the hospital into the PBPM structure.

## Conclusion

In Japan, pharmacists are now providing medication counseling to patients and information to medical staff regarding HIV treatment [6] [I. K 2006]. It is expected that PBPM will be expanded to improve HIV treatment and coordination between hospitals, pharmacies, and the community in the future. Furthermore, dosing guidance according to the patient’s history and health conditions will still be required to sustain adherence and prevent adverse effects over the long-term. It is expected that pharmacists will continue to intervene in anti-HIV treatment and take on additional responsibilities as it becomes evident that these interventions benefit patient safety and outcomes. The results of this study suggest that the implementation of PBPM improves the quality of anti-HIV drug therapy.

## Data Availability

All data generated or analyzed during this study are included in this published article.

## Abbreviations

ART: Antiretroviral therapy
CDTM: Collaborative drug therapy management
HIV: Human immunodeficiency virus
PBPM: Protocol-based pharmacotherapy management
